# This Is My Baby Interview: An Adaptation to the Spanish Language and Culture

**DOI:** 10.3390/children9020235

**Published:** 2022-02-10

**Authors:** Elena Pinero-Pinto, María-Luisa Benítez-Lugo, Raquel Chillón-Martínez, Isabel Escobio-Prieto, Gema Chamorro-Moriana, José-Jesús Jiménez-Rejano

**Affiliations:** Department of Physiotherapy, University of Seville, 41009 Seville, Spain; epinero@us.es (E.P.-P.); rchillon@centrosanisidoro.es (R.C.-M.); iescobio@us.es (I.E.-P.); gchamorro@us.es (G.C.-M.); jjjimenez@us.es (J.-J.J.-R.)

**Keywords:** interview, parents, Down syndrome, reliability

## Abstract

Evaluating the emotional state of parents is important for determining the intervention in the context of a family with a baby with Down syndrome. “This is my baby” is an interview that measures the acceptance, commitment and awareness of influence of parents towards their baby. The Spanish adaptation of this instrument helps to better understand the emotional state of parents of children with developmental disorders. A cross-cultural adaptation and reliability analysis was carried out. The results suggest that the Spanish version of the This Is My Baby interview is a reliable instrument to measure the levels of acceptance, commitment and awareness of influence of parents of an infant with Down syndrome.

## 1. Introduction

The family directly influences early childhood development in children with Down syndrome. Parents are better positioned to facilitate their children’s development, as they can maximize opportunities in everyday situations; therefore, early intervention services are integrated in the home and mediated by both parents and caregivers [1,2].

Different studies on Down syndrome performed in natural environments [1,3] demonstrate the importance of the interactions between parents and their infants with Down syndrome. Some authors [1,4,5] considered that if mother–baby interaction is promoted during early intervention by professionals, it is possible to improve the cognitive and motor development quotient of babies with Down syndrome. Parents who have followed the family-centered model during the intervention say that, by spending more time on the maturational development of their children with Down syndrome, it has been easier to accept their baby’s disability situation, as they have committed to the intervention and have realized that they can influence their baby [6]. The results of the study conducted by Mas et al. [7] indicate that the use of family-centered practices can have positive effects on the emotional well-being of parents, not only on their relationship with the baby. For all of the above, it seems that the intervention carried out from the family, and the promotion of the parent–child relationship, e.g., through infant massage, can help to improve the emotional state of the parents and the development of the baby [6,8,9,10,11].

Acceptance and commitment therapies can be included in family-focused therapies with the aim of improving the psychological state of parents, e.g., to increase their ability to modify their behavior with full awareness of their experience and context of the present moment. Therefore, the importance of rewarding values or behavior patterns, present moment awareness or mindfulness, and experiential acceptance or ongoing, open and responsive contact with the child in an emotionally challenging situation are emphasized [2]. Numerous challenges and difficulties present themselves when rearing children with developmental disorders [12]. It has been shown that mothers of babies with Down syndrome who are more dependent and need more care experience higher levels of emotional and mental health distress [13]. In general, babies need great care, thus the addition of a disability has a negative impact on maternal mental health. The readiness to receive information about their child affects the phases that parents go through, from diagnosis to acceptance of the disorder. Parental commitment during pregnancy and in the immediate months after childbirth is affected by many aspects, including happiness and emotional well-being. The greater the happiness of the parents and the better their emotional state, the greater their commitment to their children. A situation of emotional stress can affect the vulnerability of parents and decrease their commitment [14], as occurs in families with babies with Down syndrome [8]. Thus, it is important to take into account the evaluation of the emotional situation of the parents before intervening with a family in family-centered models [12], as the variables and feelings mentioned in the different phases influence the origin and persistence of family stress. Thus, Ruiz-González et al. [15] considered this when planning the intervention with the family, a fundamental aspect of which is to involve them actively.

As stated by Oppenheim et al. [16], in cases of diagnosis of pathology or serious disorder, the study of the parents’ state of mind should include not only introspection but also acceptance. In this sense, evaluations of the mental health of parents [7,17] and the impact of the arrival of a child with Down syndrome on the family [6] have been developed, although there are no evaluations of how parents accept the new situation and commit to the baby. Although parents of children with Down syndrome directly influence the development of the latter [1,4], it is known that the emotional state of the parents is a dynamic process that changes over time [12,14]. The awareness of the influence of parents of typically developing children is high in relation to their role in their children’s development [18]; however, there are no studies that describe the awareness of the influence of parents with children with Down syndrome. When analyzing the emotional state of parents with babies with Down syndrome, variables such as depression [10,19], anxiety [20,21] and resilience [3,12] are taken into account, whereas other variables are disregarded, such as acceptance, commitment and awareness of influence.

The TIMB interview was created by Bates and Dozier [22] to measure the acceptance, commitment and awareness of parental influence. It is a semi-structured interview with an approximate duration of 5–15 min. There are three versions, designed for foster, adoptive and biological parents, each with eight questions. There is only one question that differs from the rest in all three versions. Although this interview was created for foster and adoptive families, or parents with children who have a history of foster care placement, families who receive a diagnosis of a child with a disorder often reject the baby and do not accept it as their own until they are committed to and accept the situation of disability [23]. Therefore, this instrument can be used to verify whether there is a psychological conflict between the parents and the baby, in relation to acceptance, commitment and awareness of influence, which can influence their development.

However, there is no tool in the Spanish language to assess the acceptance, commitment and awareness of parental influence. These variables should be studied in families with babies with Down syndrome, as it has been shown that these variables have been described in the available scientific literature as determining aspects in the development of babies with Down syndrome. Therefore, it is necessary to adapt a specific scale to the Spanish language and culture, such as the “This Is My Baby” (TIMB) interview.

## 2. Materials and Methods

### 2.1. Design

The present study consists in the transcultural adaptation of the TIMB interview and the determination of its reliability, sensitivity to change and internal consistency. This work followed the tenets of the Declaration of Helsinki. The parents who answered the interview were informed in written and oral form about the study characteristics, benefits and risks. The participants gave their written informed consent after the nature and possible consequences of the study were explained to them. The ethics committee of the University of Seville approved this research.

### 2.2. Population and Sample

#### 2.2.1. Sample, Sampling, and Scope of Study

A total of 32 families of babies with Down syndrome participated in this multicenter study conducted in the Autonomous Community of Andalusia. The participating families were members/users of associations for people with Down syndrome and other disabilities and early childhood intervention centers. The babies of the participating families had an average age of 155.72 days (standard deviation (SD) = 39.46), with a minimum of 117 days and a maximum of 235 days. Of the 32 babies, 21 (65.6%) were boys and 11 (34.4%) were girls. In four (12.5%) of the cases, the interviewees were the fathers and in 28 cases (87.5%) they were the mothers. In addition, 17 (53.1%) of the babies had no siblings, whereas the other 15 (46.9%) had siblings.

#### 2.2.2. Selection Criteria

Inclusion criteria: families of babies with Down syndrome aged between four and eight months who received early childhood intervention (data from the babies were collected in parallel). 

Exclusion criteria: families with adopted or foster babies with Down syndrome, parents who suffer from psychiatric disorders and parents whose babies with Down syndrome suffer from associated disorders or serious illnesses (babies with Down syndrome with untreated pathologies of the heart or kidneys or digestive diseases, with another developmental disorder associated, or premature babies with Down syndrome).

A convenience non-probabilistic sampling was performed, initially contacting 43 families, of which eight did not participate, as the babies had already reached the age of eight months at the time of the interview. Three other families were subsequently excluded: two for not completing the intervention within the stipulated time, and the third one because the interview could not be conducted at the end of the intervention period.

### 2.3. Coding and Measurement Procedure

When performing any version of the TIMB interview, the tone of the interview should be conversational and should not sound like a reading, thus the questions should be memorized. Parents answer with little or no help from the interviewer, although, if the answers are short or difficult to understand, the interviewer should assist them. The use of a tape recorder is necessary for the subsequent transcribing of the answers.

The coding system consists of three indices (acceptance, commitment and awareness of influence), which reflect what the mother or father thinks about their infant and the relationship they have with him/her. The recording of the interview and its transcription allow the interviewer to classify the three indices and to codify them on a five-point Likert scale. Point and half point scores (e.g., 2.5) are accepted. The specific results are based on the weight of the positive and negative indicators of the three indices. These indicators can be the words used by the parent to describe the baby, the tone of voice used, the degree of congruence in their responses, the independence they transmit about their baby, the evidence of how important the baby is to the parent, and the experiences and examples given when endorsing their answers. Pausing to think about the answers and sharing bad experiences to back their answers tend to be negative indicators in the three indices.

#### Coding Responses into Indices

The parents were asked to develop short responses. The score is given by the content (words used), the tone of the conversation, the time to give the answer and the congruence in the answers (see Table 1 with the rubric used to code).

Examples of answers that code for “Acceptance”:

“Pablo is a lovely child; I love watching him sleep […] although sometimes I feel sorry when I look at him” (positive tone at first, but hostile tone at the end) (assigned score 3).

“This girl is too good, you know, she does nothing, she is not awake much, she eats and sleeps, she is a bit boring. But I guess these kids are like that […] as they are slower” (sad tone and long sentences, with silences) (assigned score 1).

Parents with a high level of commitment showed evidence of a strong emotional investment in the child and the consideration of the child as their own [24,25], in relation to the sense of belonging within a family and the affective parent–child bond. 

Examples of answers that code for “Commitment”:

“I don’t know what I would do without him, although he’s not what I expected” (affectionate tone, looking at the baby and smiling) (assigned score 4).

“Well, yes, I miss him if I’m not with him, but, come on … not so much yet because he was only recently born and I haven’t had time to miss him” (pauses in his/her response, listlessness in tone) (assigned score 1).

The key concept for rating awareness of influence is the degree to which the parent is predominantly focused on psychological, social or affective influences and goals as opposed to concrete influences or the achievement of physical goals [22]. 

Examples of answers that code for “Awareness of Influence”:

“I will try to give her what she needs […], we will do what we can to help Marta and make her happy […] even if it means a change of plans in the family” (positive tone, with hope and enthusiasm, quick responses) (assigned score 4).

“Well, I don’t know how we can take care of a child like that, we are not prepared for that […] I don’t know how to do this” (speaks in the third person of the baby, as if he/she were not present, hesitant) (assigned score 1)

Below are the eight interview questions corresponding to the version for biological parents [22]:

1. I would like to begin by asking you to describe (child’s name). What is (his/her) personality like?
2. If (child’s name) ever had to leave your care, how much would you miss (him/her)?
3. How do you think your relationship with (child’s name) is affecting (him/her) right now?
4. How do you think your relationship with (child’s name) will affect (him/her) in the long-term?
5. What do you want for (child’s name) right now?
6. What do you want for (child’s name) in the future?
7. Is there anything about (child’s name) or your relationship that we have not touched on that you would like to tell me?
8. I would like to end by asking a few basic questions about your experience as a parent:
  a. How many biological and/or adopted children do you have?
  b. How many biological and/or adopted children are currently living in your home?

Other studies have established acceptable levels of inter-rater reliability in the practical transcripts coded from the interviews. Twenty percent of the interviews in these previous studies were coded by both evaluators as a measure of reliability. The Spearman correlations for reliability between the evaluators (i.e., inter-rater) were 0.89 [26] and 0.90 [24]. Caregiver engagement scores ranged from 1 to 5 (M = 3.8 and standard deviation (SD) = 1.2) [26] and (M = 3.3, SD = 1.1) [24].

### 2.4. Procedure of Transcultural Adaptation to Spanish

The interview we aimed to adapt and validate is the one originally developed in English by Bates and Dozier [22] for biological parents. For the work of transcultural adaptation, the recommendations of Argimon et al. [27] were followed. The adaptation of an interview to another culture cannot be reduced to a mere literal translation but must follow a methodology that ensures the conceptual and semantic equivalence with the original and the understanding of the adapted version by the subjects. The most widely used method is translation–retranslation by bilingual people, followed by an analysis of the new version to detect discrepancies and to verify that it is understandable by a group of people.

The TIMB interview was translated into Spanish, with the prior authorization of the author of the original version, and then it was evaluated according to the following specific sequence of analysis:

1. The phenomenon to be measured was found to exist in the Spanish culture. In this sense, the definitions of acceptance, commitment and awareness of influence in Spanish and English were verified in the scientific literature, proving that, regardless of the language, the concepts did not vary and apply to numerous contexts, especially in infant psychology, adoption and disability [14].
2. The questionnaire was translated into Spanish and its conceptual equivalence was evaluated. The translation of the questionnaire was performed at two different time points, as the aim was for this translation to be rather conceptual than literal [27]. Two bilingual Spanish physiotherapists, who also knew the contents and purpose of the questionnaire, collaborated in this study, in order to prevent mistakes in the translation. From these translations, a meeting was held with the translators to agree upon the first Spanish version of the questionnaire. Subsequently, several experts in acceptance, commitment and awareness of influence evaluated the conceptual equivalence of this version with the original in English.
3. The questionnaire was retranslated into English. Once the first translated version was obtained, it was sent separately to two native bilingual English translators who specialize in health science research papers and were unaware of the original questionnaire [27]. Subsequently, a meeting was held with the translators to verify that the retranslation did not differ conceptually from the original version and thereby validate the first translation into Spanish.
4. A pilot study was carried out. This translated interview, which was considered as a draft, was submitted to a pilot study of ten fathers and mothers with babies with Down syndrome with the same characteristics as those of the families of the final sample. The aim of this pilot study was to identify confusing words or questions and to make sure that the interview questions were perfectly understandable to the mothers and fathers. During the application of this pilot study, the researchers were present to solve any doubts that the parents could have about the questions. In addition, the participants were asked to indicate those words or phrases that were confusing and give their opinion. Thus, the participants found the questions easy to understand. This did not imply any change in the wording of the questions, as no difficulties were found in the whole process of translation and retranslation. Due to the simple language used by the author of the original version, it was easy for the participants to read the questions in the study pilot. Consequently, it can be affirmed that the lexical conditions and comprehension of the items of the interview are suitable for its application.
5. Application of the interview to 32 families with babies with Down syndrome using its final version. The pertinent statistical tests were carried out to assess the concordance of the different evaluations, the interpretation of the results, the ceiling and floor effect, and the internal consistency and sensitivity. A study was carried out with these parents, with the randomization of two groups using the sealed envelope system. Sixteen parents were assigned to a group that would receive massage therapy and the other sixteen were assigned to a control group that did not receive this intervention. This massage intervention consisted of five one-hour sessions, once per week, where the parents were taught the different infant massage maneuvers [4]. The allocation to the groups was done blindly and the interviewers were also blinded to the randomization of the groups. The evaluations and interviews were carried out by different people. The test–retest was performed with the 16 parents of the control group, who received no intervention. The scores assigned to the parents after the evaluations carried out by a second expert (expert 2) to measure the test–retest reliability were agreed upon with the families who answered; this allowed the former to check the score that was assigned to their answers, based on the tone of the conversation, the words used and the time to answer, among other aspects.

### 2.5. Data Collection for the Validation of TIMB Interview

To collect the data, a researcher interviewed the parents who have agreed to participate and met the inclusion criteria. The same researcher who interviewed the parents transcribed the interviews and assigned them their index according to a Likert-type scale. Then, this researcher listened to them again, and then reassigned them a new value blindly in each scale, without taking into account the initial values, and with a time difference of one month between both evaluations. Subsequently, an expert in early care and attention to families listened to and read the transcripts of the interviews (also blindly) to give them the value that she considered at each scale. Finally, expert 2, who was familiar with the tool, interviewed, for the second time, the 16 families who did not receive any type of intervention, in order to measure the test–retest reliability of the instrument.

To understand how responses are converted into indexes, a section on specific results was included to show possible responses from parents and how they are converted into indexes, according to the coding manual of the original authors [22].

### 2.6. Data Analysis

The gathered data were analyzed using the IBM SPSS software package (SPSS Statistics 26.00, Chicago, IL, USA), Gpower 3.1.9.2 (HHU, Düsseldorf, Germany) [28] and Epidat 4.2 (Galicia Xunta, A Coruña, Spain) [29]. All statistical tests were performed considering a 95% confidence interval (*p*-value < 0.05).

First, the reliability of the Spanish version of the TIMB interview was analyzed. A description of the following data was provided: (1) the data obtained by the researcher in the evaluation performed at the beginning of the intervention (1st researcher measurement), (2) the data obtained in a second evaluation conducted on the same recordings with a difference of one month (2nd researcher measurement), (3) the data obtained by the expert evaluator and (4) the data obtained by expert 2 in two measurements with 16 subjects (test-retest). The description of these data includes the mean, standard deviation, median, first and third quartile and range scores for the three scales (acceptance, commitment and awareness of influence). For the reliability study, the inter-rater reliability between the 1st researcher measurement and the 2nd researcher measurement was analyzed. Subsequently, we assessed the consistency of the results achieved in the 1st and 2nd researcher measurements with those of the expert evaluator. The test–retest reliability was also studied in 16 subjects, comparing the values of two measurements made by expert 2. We determined the value of the intra-class correlation coefficient (ICC) [30,31]. An alpha model was used with two-way and mixed effects with absolute agreement for the intra-rater reliability, test-retest and two-way random effects with absolute agreement for inter-rater reliability [32]. To complement the ICC study, we obtained the standard error of measurement (SEM) and the minimum detectable change (MDC) [33]. In addition, the weighted Kappa coefficient was calculated employing the Cicchetti method. 

Then, the presence of ceiling and floor effects was determined. The internal consistency was evaluated by calculating the Cronbach Alpha coefficient. In each case, the value of said coefficient was verified if any of the three indices considered were eliminated (acceptance, commitment and awareness of influence). In addition, we calculated new variables, which we called “differences”, between each pair of evaluations, finding the average value of each of the difference variables and their confidence interval at 95%. In addition, the Bland and Altman graphs were performed [34].

Finally, we determined the responsiveness of the instrument, using data from a clinical trial in which 16 of the babies in our sample underwent treatment by infant massage, calculating the effect size (with the Gpower 3.1.9.2 program, HHU, Düsseldorf, Germany) of the differences found between the pre- and post-intervention values.

## 3. Results

Table 2 shows a description of the data obtained by the researcher in the 1st and 2nd measurements, those obtained by the expert evaluator in the test carried out on 32 families and those obtained in the retest carried out on 16 families by expert 2.

To analyze the consistency between the evaluations carried out in the TIMB interview, the ICC, SEM and MDC were determined for each index of the interview: (1) between the two evaluations carried out by the principal investigator, (2) between the first evaluation carried out by the investigator and the one performed by the expert, (3) between the second evaluation carried out by the principal investigator and the one implemented by the expert and (4) between the test and retest carried out by expert 2 (Table 3). As can be seen, the values obtained for the ICC show a good consistency level, with a significance of *p* < 0.001. Likewise, the SEM and MDC values decreased (Table 2). Moreover, the weighted Kappa coefficient showed values that went from moderate to excellent. Regarding the ceiling and floor effects, we must mention that the maximum and minimum possible values in each of the three indices were five points and one point, respectively. In no cases was a score of five reached for any of the three evaluations carried out (principal investigator 1st and 2nd measurement and expert measurement), and neither in the test nor in the retest, indicating the absence of ceiling effect. However, the presence of a possible floor effect (Table 2) was detected in the acceptance index in three evaluations (principal investigator measurement and expert measurement with 32 families and expert test with 16 families).

The values of Cronbach’s alpha coefficient were 0.577 and 0.647 for the first and second evaluation performed by the principal investigator, respectively, 0.739 for the one conducted by the expert, 0.564 for the test and 0.576 for the retest (*p* < 0.001). The value of Cronbach’s alpha coefficient did not increase significantly in any case when one of the three indices was eliminated.

Subsequently, the mean value of the differences between each pair of measurements and their 95% confidence interval was calculated (Table 4), observing that they were very low values.

**Figure 1:**
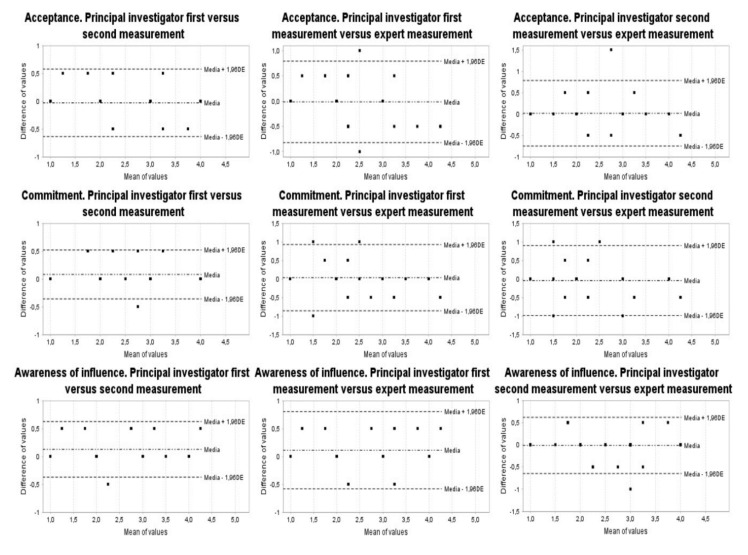
Inter-rater reliability (Bland–Altman plots).

**Figure 2:**
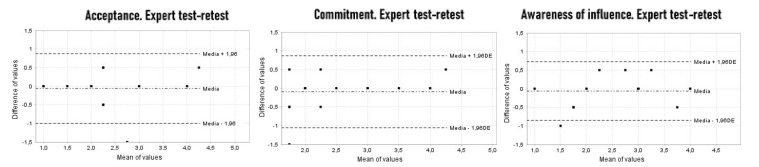
Intra-rater reliability (Bland-Altman plots).

The reliability analysis was completed by studying the Bland and Altman plots (Figure 1 and Figure 2). The mean of the two measurements analyzed is shown on the *x* axis and the difference between these two measurements is shown on the *y* axis. In addition, the mean value of the difference between the two measurements was calculated and displayed on the graph. Figure 1 and Figure 2 also show 95% limits of agreement for each comparison (average difference ± 1.96 standard deviation of the difference).

Finally, the responsiveness was studied by analyzing the effect size of the treatment with massage therapy (*n* = 16). The mean values obtained by the subjects in the TIMB interview increased in acceptance from 2.125 (SD = 0.975) to 3.875 (SD = 0.719), in commitment from 2.344 (SD = 0.944) to 3.813 (SD = 0.544), and in awareness of influence from 2.719 (SD = 0.836) to 4.000 (SD = 0.707); these increases were all statistically significant (*p* < 0.001). The size effects were 2.765, 2.613 and 1.756, respectively, indicating a very large size effect of the treatment and a good responsiveness of the TIMB interview.

## 4. Discussion

### 4.1. Concerning the Process of Choosing the Interview for Transcultural Translation and Adaptation

We believe that the choice of this interview and its extrapolation to families with infants with Down syndrome was successful. Although this interview was created for foster families, adoptive families or parents with children who have a history of foster care placement, parents with babies with Down syndrome are a population susceptible to changes in these indices after going through the different stages of grief upon receiving key information. The emotional state of parents is a cyclical, dynamic process that changes over time [3,9]. Acceptance of disability in families with babies with Down syndrome depends on multiple factors. The construction of acceptance carries with it a process of creating meaning and continuous commitment, which is developed from the interaction of parents with their baby, but also with health professionals, friends and relatives [23]. The study developed by Skotko et al. [6] points out the importance of accepting a child with Down syndrome, in addition to the fact that acceptance is one of the main life lessons these parents learn. Other studies [35,36] refer to the acceptance of parents even before birth through the prenatal diagnosis of the child with Down syndrome, although such studies do not measure this index with any tool. There is no tool in Spanish to measure the level of acceptance of parents with children with Down syndrome or any other developmental disorder. Therefore, it can be presumed that parents of children with other early-diagnosed developmental disorders could be a target population for the application of this instrument. The use of this interview in parents of children with developmental disorders can help professional teams of the family-centered model to modify practices or interventions, better adapting to the emotional needs of the family at all times [37].

We did not use the foster/adoptive parent version, as the sample consisted of biological parents, exclusively, and the difference between this interview and the biological parent version differed in a single question. Such question only refers to the parents’ previous experience in fostering or adopting. Although this interview was initially designed for adoptive, foster and biological families in a situation of separation, the results of this cross-cultural adaptation with families with children with Down syndrome seem to indicate that it is a reliable instrument to measure acceptance, commitment and influence awareness.

### 4.2. Concerning the Transcultural Translation and Adaptation Process

We consider that it constitutes a qualitative methodology accepted for the transcultural adaptation of an interview, as performed by Martínez et al. [38]. The results of Martínez et al. are in line with our results in the initial translation and re-translation process, as well as in the internal consistency of the instrument according to the qualitative research strategies defined by Patton [39], Guba and Lincoln [40], and Denzin and Lincoln [41].

Robles et al. [42] analyzed their interview in the same way as in the present study. Firstly, they analyzed the internal consistency, through Cronbach’s Alpha coefficient, which in their case was 0.915. To verify the reliability of the instrument, they also interviewed a group of five people, with a time interval of two weeks, resulting, in this case, in an ICC of 0.894 (*p* < 0.01). In our study, the final version of the interview was applied to 32 families with babies with Down syndrome; these results were evaluated on two occasions by the principal investigator, with a time difference of one month, obtaining ICC values that show a good consistency level, with a significance of *p* < 0.001. In the case of Parra [43], he analyzed the interview’s inter-dependence reliability, which is the coincidence between the judges regarding the value given to each index or category, through the Kappa concordance coefficient. In our interview, the same process was carried out through the analysis of the two evaluations performed by the principal investigator with respect to the evaluation carried out by the external expert. In this case, the average value of Kappa was 0.1358, indicating that this instrument does not consistently produce equivalent results among the judges. However, in our case, the weighted Kappa coefficient showed values that went from good to excellent, which indicates that there is coincidence or inter-trial reliability in our study.

### 4.3. Concerning the Reliability of the TIMB Interview Adapted to the Spanish Language

For the reliability of our instrument, we carried out a study of the inter-rater reliability with the data obtained by each index of the interview in the two evaluations carried out by the principal investigator and, on the other hand, between the evaluations made by the investigator and the expert evaluator. The results showed a good consistency level, with a significance of *p* < 0.001, according to the criteria of Trop et al. [44] and Pita et al. [45]. This confirms the high degree of concordance between the two evaluations on the initial results of the principal investigator.

The calculation of the ICC for each index of the interview showed very high results intra-rater reliability between the two evaluations performed by the investigator and between the first one carried out by the investigator and the one carried out by the expert evaluator (all above 0.9). This leads us to establish that the transcultural adaptation of the TIMB interview to Spanish and the obtained results are valid. The results of the two evaluations of the principal investigator and that of the expert indicate that, for the acceptance index, there was a possible floor effect. This may be due to the emotional impact on families with infants with Down syndrome in the first months of life, which evolves towards acceptance and commitment to their infant as they relate to him/her [12]. Regarding the test and retest carried out by expert 2, the values obtained for the ICC show a good consistency level, with a significance of *p* < 0.001. Likewise, the SEM and MDC values decreased, and the inter and intra-examiner reliability provide good results, indicating that this tool is reliable when used by the same observer or by different observers. 

The three indices analyzed in the interview have no direct relationship with each other; in fact, each of them was analyzed independently, which would explain the results of the Cronbach’s alpha coefficient values.

#### 4.3.1. Limitations

The limitations derived from this research are defined by several factors. First, the results obtained with these instruments have limitations when used with parents of children with Down syndrome, as they do not address other variables, such as grief or denial, as processes.

On the other hand, this interview has not been used, until now, with families with children with developmental disorders. It is necessary to investigate other developmental disorders and carry out further research with parents with children with Down syndrome throughout the national territory in order to generalize the results, as a sample has only been obtained from part of the Spanish population. More validity studies of the TIMB interview are required, with parents of children with typical development, as well as with other developmental disorders, in order to guarantee the widespread use of the tool in other populations. 

The interview was translated, but cultural specificity regarding the content of the interview was not assessed. It would be interesting to study if there are cultural differences in the emotional state of parents and/or their expression.

Lastly, the sample size may have been limited due to the fact that recruiting was conducted only once.

#### 4.3.2. Future Research and Practical Relevance

In the future, it would be interesting to study the perception that parents have about their levels of acceptance, commitment and awareness of influence, and compare it with the results obtained in the interview. It would also be interesting to carry out different measurements to check the test–retest reliability of the instrument.

The adaptation of the This Is My Baby interview implies an increase in the range of possibilities for the initial evaluation or screening of families with children with Down syndrome. The TIMB interview can serve as a screening instrument to detect problematic situations in families and, if this occurs, they can be evaluated in depth by psychologists, as well as taken into account to carry out the family-centered approach. The family-centered model requires the active participation of parents in the intervention. This means that parents must want to participate in it, and if the emotional situation is not positive, it will be difficult for them to integrate into the intervention process. In addition, it has been shown that parents are more committed when their emotional state is good, which also conditions the development of their babies.

## 5. Conclusions

The Spanish adaptation of the TIMB interview was a reliable instrument to measure the levels of acceptance, commitment and awareness of influence of the parents of infants with Down syndrome of our sample. Studies with a larger sample size are necessary to generalize our results to the entire population of parents with children with Down syndrome.

## Figures and Tables

**Table 1:** Rubric to code the parents’ responses on the Likert scale for the indices of acceptance, commitment and awareness of influence.

		1	2	3	4	5
Acceptance	Words and content	Rejection or disgust when talking about the child	Negative maternal feelings	No positive or negative words, or mixed words	Relationship is positive	Describes relationship as very positive
	Tone of conversation	Very hostile tone	Hostile tone	Medium	Positive tone	Very positive tone
	Time to give the answer	Very large	Large	Medium	Quick answer	Very quick answer
Commitment	Words and content	Talks about the child as if he/she were not his/hers	Little feeling of belonging	Mixed words	Feelings of belonging	Obvious feelings of belonging
	Tone of conversation	No efforts to form an affective bond	Little efforts to form an affective bond	Mixed tone during interview	Active efforts to form an affective bond	Very active efforts to form an affective bond
	Time to give the answer	Very large (too many pauses)	Large	Medium	Quick answer	Very quick answer
Awareness of influence	Words and content	Does not show that he/she influences the baby	Some sign about his/her influence	Mixed content	Promotes the child’s sense of being loved or feeling secure	Promotes the development of age-appropriate psychological autonomy
	Tone of conversation	Great hostile tone, speaking of the baby in the third person	Hostile tone	Mixed tone	Loving tone	Great loving tone, of affection
	Time to give the answer	Very large (too many pauses)	Large	Medium	Quick answer	Very quick answer

**Table 2:** Description of the data obtained in the evaluations carried out by the principal investigator (first and second measurements), those obtained by the expert evaluator and those obtained in the test and retest.

	Index	Mean	Standard Deviation	Median	1st–3rd Quartile	Range	Floor Effect (% Value 1)
Principal investigator—1st measurement *n* = 32	Acceptance	2.172	0.921	2.0	1.250–3.0	1.0–4.0	25
	Commitment	2.453	0.836	2.0	2.0–3.0	1.0–4.0	9.4
	Awareness of influence	2.688	0.849	3.0	2.0–3.0	1.0–4.50	3.1
Principal investigator–2nd measurement *n* = 32	Acceptance	2.203	1.007	2.0	1.0–3.0	1.0–4.0	28.1
	Commitment	2.375	0.852	2.0	2.0–3.0	1.0–4.0	9,4
	Awareness of influence	2.563	0.849	2.5	2.0–3.0	1.0–4.0	9.4
Expert test *n* = 32	Acceptance	2.188	1.045	2.0	1.0–3.0	1.0–4.50	28.1
	Commitment	2.422	0.993	2.0	1.750–3.0	1.0–4.50	9.4
	Awareness of influence	2.578	0.881	3.0	2.0–3.0	1.0–4.0	9.4
Expert test *n* = 16	Acceptance	2.188	1.047	2.0	1.250–2.750	1.0–4.50	25.0
	Commitment	2.438	0.947	2.0	2.0–3.0	1.0–4.50	6.3
	Awareness of influence	2.531	0.921	3.0	1.750–3.0	1.0–4.0	12.5
Expert retest *n* = 16	Acceptance	2.250	1.032	2.0	1.250–3.0	1.0–4.0	12.5
	Commitment	2.531	0.763	2.25	2.0–3.0	1.5–4.0	3.1
	Awareness of influence	2.594	0.800	2.75	2.0–3.0	1.0–4.0	3.1

**Table 3:** Intra-class correlation coefficient, standard error of measurement (SEM), minimum detectable change (MDC) and significance of the evaluations carried out by the principal investigator (first and second measurements) and the expert and those obtained in the test and the retest.

	Index	ICC	*p*-Value	SEM	MDC	Weighted Kappa
Principal investigator—1st measurement Principal investigator—2nd measurement *n* = 32	Acceptance	0.950	<0.001	0.219	0.607	0.824
	Commitment	0.962	<0.001	0.158	0.438	0.878
	Awareness of influence	0.946	<0.001	0.179	0.496	0.832
Principal investigator—1st measurement Expert test *n* = 32	Acceptance	0.915	<0.001	0.291	0.807	0.753
	Commitment	0.879	<0.001	0.322	0.894	0.688
	Awareness of influence	0.912	<0.001	0.249	0.690	0.723
Principal investigator—2nd measurement Expert test *n* = 32	Acceptance	0.929	<0.001	0.276	0.765	0.820
	Commitment	0.867	<0.001	0.341	0.944	0.673
	Awareness of influence	0.932	<0.001	0.228	0.632	0.820
Expert test-retest *n* = 16	Acceptance	0.898	<0.001	0.646	1.792	0.777
	Commitment	0.841	<0.001	0.657	1.821	0.688
	Awareness of influence	0.895	<0.001	0.544	1.507	0.733

**Table 4:** Mean values of the differences between each pair of evaluations and their confidence interval at 95%.

Comparison	Difference in the Index	Mean	Confidence Interval at 95%
			Lower Limit/Upper Limit
Principal investigator—1st measurement Principal investigator—2nd measurement *n* = 32	Acceptance	−0.0313	−0.1428/0.0803
	Commitment	0.0781	−0.0026/0.1589
	Awareness of influence	0.1250	0.0334/0.2166
Principal investigator—1st measurement Expert *n* = 32	Acceptance	−0.0156	−0.1639/0.1326
	Commitment	0.0313	−0.1334/0.1959
	Awareness of influence	0.1094	−0.0180/0.2367
Principal investigator—2nd measurement Expert *n* = 32	Acceptance	0.0156	−0.1254/0.1566
	Commitment	−0.0469	−0.2204/0.1266
	Awareness of influence	−0.0156	−0.1322/0.1010
Expert test-retest *n* = 16	Acceptance	−0.0625	−0.3176/0.1926
	Commitment	−0.0938	−0.3551/0.1676
	Awareness of influence	−0.0625	−0.2773/0.1523

## Data Availability

The data presented in this study are available on request from the corresponding author.
